# Alternative Approaches for the Management of Diabetic Foot Ulcers

**DOI:** 10.3389/fmicb.2021.747618

**Published:** 2021-10-05

**Authors:** Cassandra Pouget, Catherine Dunyach-Remy, Alix Pantel, Adeline Boutet-Dubois, Sophie Schuldiner, Albert Sotto, Jean-Philippe Lavigne, Paul Loubet

**Affiliations:** ^1^Virulence Bactérienne et Infections Chroniques, INSERM U1047, Université de Montpellier, Nîmes, France; ^2^Virulence Bactérienne et Infections Chroniques, INSERM U1047, Université de Montpellier, Service de Microbiologie et Hygiène Hospitalière, Clinique du Pied Gard Occitanie, CHU Nîmes, Nîmes, France; ^3^Virulence Bactérienne et Infections Chroniques, INSERM U1047, Université de Montpellier, Service des Maladies Métaboliques et Endocriniennes, Clinique du Pied Gard Occitanie, CHU Nîmes, Le Grau-du-Roi, France; ^4^Virulence Bactérienne et Infections Chroniques, INSERM U1047, Université de Montpellier, Service des Maladies Infectieuses et Tropicales, Clinique du Pied Gard Occitanie, CHU Nîmes, Nîmes, France

**Keywords:** alternative therapeutic approaches, biofilm, chronic wound, diabetic foot, antibiofilm

## Abstract

Diabetic foot ulcers (DFU) represent a growing public health problem. The emergence of multidrug-resistant (MDR) bacteria is a complication due to the difficulties in distinguishing between infection and colonization in DFU. Another problem lies in biofilm formation on the skin surface of DFU. Biofilm is an important pathophysiology step in DFU and may contribute to healing delays. Both MDR bacteria and biofilm producing microorganism create hostile conditions to antibiotic action that lead to chronicity of the wound, followed by infection and, in the worst scenario, lower limb amputation. In this context, alternative approaches to antibiotics for the management of DFU would be very welcome. In this review, we discuss current knowledge on biofilm in DFU and we focus on some new alternative solutions for the management of these wounds, such as antibiofilm approaches that could prevent the establishment of microbial biofilms and wound chronicity. These innovative therapeutic strategies could replace or complement the classical strategy for the management of DFU to improve the healing process.

## Introduction

Diabetic foot ulcers (DFU) have a lifetime prevalence of 15–25% (Armstrong et al., 2017). Infection is the most common, severe and costly (Prompers et al., 2008) DFU complication with high risk of mortality and morbidity associated with lower limb amputation (Bakker et al., 2016). The diagnosis of diabetic foot infection (DFI) is often difficult, leading to the inappropriate use of antibiotics. The bacterial organization in DFU and the involvement of multidrug-resistant (MDR) bacteria require new antimicrobial solutions. This review discusses the role of the biofilm in DFU and alternative approaches to classical treatment that could improve DFU management.

### Clinical and Translational Relevance

Sixty to 80% of chronic wounds harbor bacterial structures in a biofilm (James et al., 2008; Malone et al., 2017a). For the clinician, the main difficulty is to distinguish between infecting and colonizing bacteria. Misclassification can lead to inappropriate antibiotic prescriptions that contribute to promoting the emergence of MDR bacteria, a major DFU health issue (Caravaggi et al., 2013). Better understanding of the bacterial organization of biofilms in chronic wounds would allow development of tailored antimicrobial strategies and improving wound healing. In this context, a large majority of current fundamental studies on DFUs focuses on bacterial cooperation and the impact of local microenvironment on microorganisms. Thus, the host-microorganism interface plays a major role in DFI development. In DFU, bacteria are classically organized in functionally equivalent pathogroups (FEP) where pathogenic and commensal bacteria co-aggregate symbiotically in a pathogenic biofilm to maintain a chronic infection (Dowd et al., 2008). Polymicrobial biofilms have been observed both in pre-clinical studies using animal models and in clinical research on DFU. They represent the main cause of healing delay. Recently, some approaches have targeted biofilm formation with the aim of controlling infections (Snyder et al., 2017). Better understanding of the host-bacterial interactions is essential to develop new therapeutic solutions that take into account the biofilm to limit the diffusion of MDR bacteria.

### Diabetic Foot Ulcers and Biofilms

Biofilm formation is a multistep process (see for review Percival et al., 2015) whereby heterogeneous communities of microorganisms (bacteria and/or fungi) are embedded into an extracellular polymeric substance (EPS) matrix that contains proteins, deoxyribonucleic acid (DNA), glycoproteins and polysaccharides, and confers the ability to adhere to biotic or abiotic surfaces (Bjarnsholt, 2013). In DFU, the biofilm architectural structure differs among patients due to the variability of the involved bacterial genera and species. Conversely, the multistep formation process is similar. Biofilm formation is a major mechanism of adaptation that protects bacteria from antibiotics, due to several characteristics (Singh et al., 2017). Biofilm structure provides a protective layer against antimicrobial compounds. Wounds biofilms are polymicrobial, formed by complex and order combinations of microorganisms. Hence, compounds produced by different bacterial strains might impair the contact between the bacterial cell wall and the antibiotic by changing the composition of the EPS. Finally, the production of degradative enzymes by different pathogens can act in synergy against antibiotics. These biofilm aspects are responsible for a reduced diffusion of the antibiotic within the biofilm matrix leading to an inefficient activity of the antibiotic treatment (Sharma et al., 2019). In addition to this feature, the ability to form a biofilm is an effective strategy to enhance survival and persistence of microorganisms by increasing their antimicrobial resistance. The antimicrobial resistance in organisms producing biofilms acts by delayed penetration of the antimicrobial agents through the biofilm matrix, altered growth rate of biofilm organisms, and other physiological changes due to the biofilm mode of growth (Donlan and Costerton, 2002).

## Classical Strategies in the Management of Diabetic Foot Ulcers

The management of patients with a DFU is a multidisciplinary approach that includes all relevant specialties (i.e., nursing, orthopedics, plastic surgery, vascular surgery, nutrition, infectious diseases, microbiology, and endocrinology departments) (Cahn et al., 2014). To aid the clinician during the management of DFUs, classification of stage and severity of the wound must be established (Lipsky et al., 2020). The classical care for the control and treatment of DFUs is centered on perfusion, pressure moderation, control of the infection, control of the glycaemic balance, foot discharge and debridement (Wu et al., 2007) (Table 1).

**TABLE 1:** *In vitro* and *in vivo* effects of the main alternative approaches studied.

	***In vitro* effects**	***In vivo* effects**	**References**
**Debridement**			
Negative pressure therapy	**–**	Enhance wound closure	Apelqvist et al., 2017; Liu et al., 2018
**Antimicrobial agents**			
Calcium sulfate beads with antibiotics	Decreased viability of MRSA strains	No clinical evaluation	Price et al., 2016
Nanoparticles	Silver nanoparticles affect *P. aeruginosa* biofilm formation	No clinical evaluation	Beyth et al., 2015; Ahmadi and Adibhesami, 2017; Hamdan et al., 2017; Mihai et al., 2018
Oxyclozanide	Enhances aminoglycoside and tetracycline killing in *S. aureus* biofilms	No clinical evaluation	Maiden et al., 2019
Guanylated polymethacrylates	Effective killing of *C. albicans* and *S. aureus* in polymicrobial biofilms	Untested in human DFU	Qu et al., 2016
Guar gum-associated nisin	Reduction of biofilm formation by *S. aureus* isolates from patients	Evaluation with strains isolated from DFI	Cirioni et al., 2006; Dutta and Das, 2016; Santos et al., 2016; Thombare et al., 2016
Acapsil	–	Shorter hospital stay and faster wound healing	Bilyayeva et al., 2017
**Antiseptics**			
Cadexomer iodine	**–**	Reduction (1 log10) of microbial load and biofilm in DFU (11/17 patients)	Schwartz et al., 2013; Malone et al., 2017b
**Nutraceuticals**			
Cranberry	Inhibition of pilus synthesis and prevention of biofilm formation	Decrease of *Escherichia coli, S. aureus* adhesion	LaPlante et al., 2012
Tannic acid	Inhibition of *S. aureus* biofilm formation by peptidoglycan cleavage	Acceleration of cutaneous wound healing in rat model	Payne et al., 2013; Orlowski et al., 2018; Chen et al., 2019
Tea-tree oil and Cinnamon oil	Effect on MRSA biofilm	Reduction of the quantity of colonized MRSA and promotion of healing of chronic wounds in a clinical trial	Kwieciński et al., 2009; Lee et al., 2014; Cui et al., 2016; Seyed Ahmadi et al., 2019
Ellagic acid	Limits *S. aureus* biofilm formation and enhances antibiotic susceptibility	No clinical evaluation	Quave et al., 2012
Propolis and honey	Anti-inflammatory and anti-bacterial properties	Reduction of bacterial load of chronic wounds in combination with antibiotics	Henshaw et al., 2014; Jull et al., 2015; Martinotti and Ranzato, 2015; Minden-Birkenmaier and Bowlin, 2018; McLoone et al., 2020
Probiotics	*Lactobacilli* antibiofilm activity	Acceleration of wound healing in mice	Vuotto et al., 2014; Vågesjö et al., 2018
**Phage therapy**			
	Reduction of biofilm formation and infection by *P. aeruginosa*, *S. aureus*, and *A. baumannii*	Reduction of bacterial load and wound closure in diabetic mouse wound infections	Mendes et al., 2014; Fish et al., 2018; Hill et al., 2018; Morozova et al., 2018; Taha et al., 2018; Albac et al., 2020; Kifelew et al., 2020
**Action on wound healing**			
Photodynamic therapy	**–**	Increase of reepithelization	Tardivo et al., 2014
Hyperbaric oxygen therapy	**–**	Improvement of short-term healing	Kranke et al., 2015
Non-thermal plasma	**–**	Acceleration of wound healing in animal models of ulcers	Chatraie et al., 2018; Cooley et al., 2020
Electrostimulation	Enhanced wound closure time	Evaluation with dressings	Barki et al., 2019
**Alternatives for inhibition of adhesion and biofilm**			
**Inhibition of initial bacterial adhesion**			
EDTA and citrate	Prevention of biofilm formation and degradation of pre-existing biofilm (via Mg^2+^, Ca^2+^, and iron chelators)	Prevention of infection in a rabbit catheter model (with minocycline)	Raad et al., 2008
Aryl rhodanines	Inhibition of biofilm formation by *S. aureus* and other Gram-positive bacteria by targeting early stage of adhesion	No clinical evaluation	Opperman et al., 2009
**Interaction with biofilm metabolism by QS stimulus modulation**			
Furanone	Inhibition of biofilm formation and expression of *P. aeruginosa* virulence factors	Decrease of *P. aeruginosa* virulence	Kim et al., 2012; García-Contreras et al., 2013
Sodium ascorbate	Modulation of QS signal in *P. aeruginosa*	No clinical evaluation	El-Mowafy et al., 2014
Savarin	Inhibition of *S. aureus* biofilm formation (by targeting *agr*)	No clinical evaluation	Sully et al., 2014
Azithromycin	Inhibition of biofilm formation and expression of *P. aeruginosa* virulence factors	Improvement of clinical signs in patients with CF and *P. aeruginosa* infections	Bala et al., 2011
RNAII inhibiting peptide	Reduction of *S. aureus* virulence	Healing improvement in a chronic wound mouse model	Giacometti et al., 2003
c-di-GMP	Reduction of biofilm formation in *P. aeruginosa* and *A. baumannii*	No clinical evaluation	Romling et al., 2013; Lieberman et al., 2014; Wu et al., 2015
Exo-polysaccharides	Reduction of biofilm formation (*P. aeruginosa*) by targeting virulence factors + PA01 and *S. epidermidis* in co-culture	No clinical evaluation	Pihl et al., 2010; Jiang et al., 2011; Rendueles et al., 2013; Limoli et al., 2015
1,018-peptide and derivates	Disruption of *P. aeruginosa* and *B. cenocepacia* mature biofilms	No clinical evaluation	Willcox et al., 2008; de la Fuente-Núñez et al., 2012, 2014
Deferiprone	Activity against coagulase-negative staphylococci	No clinical evaluation	Coraça-Huber et al., 2018
**Enzymes enhancing bacterial dispersion**			
α-amylase	Disruption of biofilm formed by *S. aureus*	No clinical evaluation	Kalpana et al., 2012
α-amylase and cellulase	Disruption of biofilm	*In vivo* disruption but the dispersal can cause systemic infection	Fleming et al., 2017
DNase, dispersin B	Eradication of single and multi-species biofilms	No clinical evaluation	Chen and Lee, 2018; Sharma and Pagedar Singh, 2018
2-aminoimidazole	Disruption of biofilms formed by *S. aureus*	No clinical evaluation	Rogers et al., 2010
Lysostaphin	Eradication of *P*. *aeruginosa* biofilms	Effective treatment for biofilm disruption on jugular vein catheters in mice	Kokai-Kun et al., 2009
C2DA	Dispersion of *S. aureus*, Action on MRSA biofilm	No clinical evaluation	Jennings et al., 2012
**Next-generation dressings and grafts**			
NGAD NGAD + mesenchymal stem cells	Removal of biofilms by *S. aureus* and antibiotic-resistant *P. aeruginosa*	Evaluation with clinical strains	Parsons et al., 2016; Pérez-Díaz et al., 2018; Tarusha et al., 2018
Electrospun nanofibers	Prevent biofilm formation and enhance fibroblast development	No clinical evaluation	Ramalingam et al., 2019
Surfactant based gel	–	Reduced bacteria development and biofilm infection	Yang et al., 2017; Percival et al., 2018
Dehydrated amniotic membranes	Faster wound healing in patients with severe comorbidities	Lower extremity wounds	Lullove, 2017
Sucrose octasulfate	–	Significant increase of wound closure rate	Edmonds et al., 2018
Skin substitutes	–	Fish skin offers natural anti-inflammatory properties and promotes growth of new skin. Other wounds and patients with burns	See clinicaltrials.gov NCT01348581
*Arenicola marina*	This new dressing delivers oxygen to the wound bed, enhancing healing and cell proliferation	No evaluation clinical	Le Pape et al., 2018
Epigel^®^	This new bioactive hydrogel hydrates the wound bed	No clinical evaluation	See www.epinovabiotech.com
Keratinocyte treatment. Skin grafts (epithelial or fetal cells). Stem cells. Collagen I matrix. Human placental tissues.	–	Improve closure time	Kanji and Das, 2017; Lo et al., 2019; Lintzeris et al., 2018; Mao et al., 2018; Momeni et al., 2019; Hassanshahi et al., 2019; Hwang et al., 2019; Oropallo, 2019
3D-printed scaffolds	–	Shorter healing time	Pushparaj and Ranganathan, 2017; Sun et al., 2018

*EDTA, ethylene diamine tetra-acetic; EGTA, egtazic acid; MRSA, methicillin-resistant *Staphylococcus aureus*; QS, quorum sensing; CF, cystic fibrosis; C2DA, *cis*-2-decenoic acid; DFI, diabetic foot infection; DFU, diabetic foot ulcer; NGAD, next-generation carboxymethylcellulose silver- containing wound dressing.*

### Debridement of the Wound

Debridement consists in the removal of necrotic, devitalized and/or infected tissue from a wound, leaving healthy tissue preserved. Surgical debridement is the usual method used. The objective is to control the bacterial load, which, in combination with antimicrobial treatment, allows early closure of the wound (Wolcott et al., 2009). Debridement enables the wound and surrounding tissues to promote normal healing by removing infected tissues, biofilms, and senescent cells. Debridement allows the reepithelialization of soft-tissue by eradication of (early or established) infection and reduction of bioburden, the improvement of local blood flow, and the revitalization of the wound bed. When it was performed correctly, it optimizes the diabetic wound healing.

### Negative Pressure Wound Therapy

Associated with debridement, negative pressure wound therapy is an airtight open-pore placed onto the wound and covered by an airtight dressing. Then, the wound is connected to a vacuum source and a negative pressure is generated. The negative pressure at the wound site reduces the size of the wound through contraction; it continuously cleans the wound by removing small debris through suction and reduces levels of proteases through wound fluid removal (Apelqvist et al., 2017). The efficiency of this therapy has been confirmed and it represents an effective measure of promoting wound healing, although the evidence is low (Liu et al., 2018).

### Antimicrobial Therapy for Infected Diabetic Foot Ulcers

Antibiotics are not used to manage colonized DFU (Lipsky et al., 2020). Their use concerned the different stages of DFI. Antibiotics are mainly empirical in the first instance, in accordance with the causative pathogen and the severity of the infection. The definitive antibiotic treatment is changed according to the microbiological culture and the response of the empirical treatment (Kwon and Armstrong, 2018). Its duration will depend on the severity of the infection. However, Walker et al. (2015) reported that 74% of DFUs did not respond to topical and systemic agents. Recently, Johani et al. (2018) confirmed this observation. Uçkay et al. (2018) could not demonstrate a beneficial effect of topical therapy using gentamicin-sponges in 88 DFUs. Similar conclusions were drawn for vancomycin powder, although infections were more superficial in patients treated with vancomycin than in controls (untreated) (Wukich et al., 2015). A recent Cochrane review on this topic indicated that randomized controlled data on the effectiveness and safety of topical antimicrobial for DFI are limited (Dumville et al., 2017).

As bacteria in biofilms display 100 to 1,000-fold higher tolerance to antibiotics, new solutions to deliver antibiotics at high concentration into the biofilm have been developed. Delivery systems could be used to administer high concentrations of antibiotics to the wound with limited side effects. Biodegradable vehicles, such as calcium sulfate beads, display a good elution profile and seem to be compatible with many antibiotics. Natural polymers, such as collagen sponges, are another emerging delivery system, although data are still limited for DFU (Markakis et al., 2018). For instance, calcium sulfate beads are mineral elements that are naturally absorbed into biofilms and then slowly dissolve to release antibiotics. Price et al. (2016) showed *in vitro* that calcium sulfate beads loaded with gentamicin or tobramycin eradicated *Pseudomonas aeruginosa* biofilms in DFU, and also reduced the viability of MRSA strains. The main problem of this approach is the potential risk of bacterial resistance selection. Randomized trials are required to confirm the efficacy of these approaches.

Recently, Maiden et al. (2019) showed that the ionophore oxyclozanide can enhance aminoglycoside and tetracycline killing activity in *P. aeruginosa* biofilms by reducing the bacterial cell membrane potential and increasing antibiotic accumulation within the biofilm. Currently, this compound is mainly used in veterinary medicine for parasitic infections, but this finding suggests that in combination with aminoglycosides, oxyclozanide could represent a new antibiofilm agent for chronic wound treatment.

## Alternative Approaches in the Management of Diabetic Foot Ulcers

In addition to conventional approaches, new alternative solutions have emerged in recent years, targeting the bacterial organization and notably the biofilm formation of DFU (Table 1).

### Antimicrobial Peptides and Related Drugs

Santos et al. (2016) reported that nisin, a bacteriocin against Gram-positive bacteria, was active against some Gram-negative bacteria. Nisin promotes the disintegration of the bacterial cell membrane lipid bilayer by electrostatic interactions. Its use in DFU requires an effective delivery system. An *in vitro* study showed that guar gum-associated nisin reduced biofilm formation by 23 *S. aureus* strains isolated from DFU, including MDR strains (Thombare et al., 2016). Similarly, citropin is active against *P. aeruginosa* and *S. aureus* without major toxicity in animal models (Cirioni et al., 2006). However, Dutta and Das (2016) highlighted the limitations of antimicrobial peptides (AMPs), especially in terms of production costs, bioavailability, and difficult clinical translation.

Guanylated polymethacrylates are a new class of antimicrobial agents that structurally mimics AMPs and efficiently kills both fungi and bacteria in polymicrobial biofilms (*Candida albicans* and *S. aureus*) (Qu et al., 2016). A study on 266 patients with venous leg ulcers and DFU showed that, compared with gentaxane and iodine/dimethyl sulfoxide (DMSO), Acapsil^®^ (Willingsford Healthcare), a powder based on a micropore particle technology, accelerated wound healing and reduced hospitalization length (Bilyayeva et al., 2017).

### Nanotechnologies

Nanotechnology-based therapies open the door to new therapeutic solutions for chronic wounds (Hamdan et al., 2017). Nanoparticles made of iron, silver, zinc, or titanium showed antibacterial activity (disruption of the bacterial membrane) (Beyth et al., 2015), and due to their high bioavailability, they can penetrate into mature biofilms and target sessile bacteria. Therefore, these materials could be used to target both surface bacteria and biofilm-organized bacteria in deeper tissues. A recent study showed that the combination of silver nanoparticles and tetracycline reduced the bacterial load and promoted healing in wounds inoculated with *P. aeruginosa* in mice (Ahmadi and Adibhesami, 2017). A recent review has summarized nanotechnology-based wound healing approaches and their benefits (Mihai et al., 2018).

### Antiseptics

Topical antiseptics are antimicrobial agents that inhibit or reduce the number of microorganisms. Unlike antibiotics, antiseptics have multiple targets and a broader spectrum of activity including bacteria, fungi, viruses, or protozoa. They have commonly been used on wounds to prevent or treat infection; however, antiseptic fluid irrigation have received little scientific study and their efficiency remain questioned (Lipsky et al., 2020). Indeed, wound cleansers may affect normal human cells and may be antimitotic affecting normal tissue repair. Repeated and excessive treatment of wounds with antiseptics without proper indications may have negative outcomes or promote a microenvironment similar to those found in chronic wounds. With the discovery of polymicrobial biofilms and the emergence of bacteria tolerant to antiseptics, their effectiveness is even more questionable (Sheldon, 2005; Ortega Morente et al., 2013; Stewart, 2015). Following these observations, international guidelines suggest that antiseptics are not appropriate in the management of DFU (Lipsky et al., 2020).

However, some recent studies present interesting results. Products, such as Octenilin^®^ (Schülke & Mayr GmbH), iodine-based solutions, polyhexamethylene biguanide or silver-impregnated dressings, are good *in vitro* candidates (Kucisec-Tepes, 2016; Pavlik et al., 2019) to reduce biofilms, but their effectiveness against polymicrobial and complex biofilms remains to be demonstrated (Khan and Naqvi, 2006). Similarly, chlorhexidine action is clearly limited on multi-species biofilms (Touzel et al., 2016). Townsend et al. (2016) developed a new *in vitro* inter-kingdom wound biofilm model on hydrogel-based cellulose to test the efficacy of common topical antiseptics. They treated biofilms composed of *C. albicans, P. aeruginosa*, and *S. aureus* with chlorhexidine or povidone iodine, and found that the structure of polymicrobial biofilms was only slightly affected compared with that of monomicrobial biofilms. They also showed that topical antiseptics were less efficient against polymicrobial biofilms.

Cadexomer iodine is a topical antimicrobial agent that could be used to deliver iodine into wounds. Iodine can penetrate the pathogen cell wall and disrupt proteins, as well as the nucleic acid structure and synthesis. Cadexomer iodine can be encapsulated within small polysaccharide beads that, in the presence of the wound exudate, start to swell and release iodine into the wound. *In vivo* studies have demonstrated that cadexomer iodine significantly reduced biofilm and microbial load in DFU (Schwartz et al., 2013; Malone et al., 2017b).

### Nutraceuticals

Nutraceuticals are pharmaceutical alternatives that include all foods or food products which provide medical benefits and can be delivered under medical form. These products could present health benefits, and several plant-derived natural compounds could prove clinically beneficial.

A study has reported that cranberry extracts inhibited biofilm production of Gram-positive bacteria (LaPlante et al., 2012). Polyphenolic compounds, such as tannic acid (Orlowski et al., 2018; Chen et al., 2019 in a rats model) and tea-tree oil (Lee et al., 2014 in a clinical trial), also inhibited biofilm formation by *S. aureus*, including methicillin-resistant *S. aureus* (MRSA) (Kwieciński et al., 2009), by cleaving peptidoglycan (Payne et al., 2013). An active compound found in cinnamon oil has also been shown to prevent MRSA biofilm formation *in vitro* (Cui et al., 2016), and also in a mice model of wound infection (Seyed Ahmadi et al., 2019). Finally, ellagic acid derivatives also limited *S. aureus* biofilm formation and enhanced its susceptibility to some antibiotics (Quave et al., 2012). All these compounds must be clinically evaluated in chronic wounds.

The natural anti-inflammatory and antimicrobial properties of propolis produced by honeybees are well known. Its regenerative properties and low cost explain the increased interest in propolis for promoting chronic wound healing (Henshaw et al., 2014; Martinotti and Ranzato, 2015). To our knowledge, propolis alone has not been used in DFU, but a recent review summarized the effect of propolis with a combination of several antibiotics in skin problems including wounds (McLoone et al., 2020).

Honey has been used for a long time to treat wounds with no real proof of its efficiency. A study demonstrated that in animals, honey has a clear antibacterial effect, but no anti-inflammatory activity (Jull et al., 2015). Recently, Minden-Birkenmaier and Bowlin (2018) reviewed the effects of different types of honey on wound closure and antibiofilm activity. They also discussed the advantages of honey in the field of tissue engineering and biomaterials (Cryogels, Electrospun templates, and Hydrogels).

Within a biofilm, intra- and inter-species interactions and cooperation can be observed at the different stages of its formation. Another approach could be to harness the bacterial competition to modify the dispersion or modification of the growing matrix. Probiotic bacteria, such as Lactobacilli, could have antibiofilm activities and be good candidates for wound treatment (Vuotto et al., 2014; Vågesjö et al., 2018 in *in vivo* model).

### Phage Therapy

There is renewed interest in bacteriophages to fight bacteria. In this treatment, viruses infect a specific bacterium and reproduce inside it. Several areas must be investigated to evaluate the potential of bacteriophages in the therapeutic arsenal (Knezevic et al., 2021). Indeed, the success of phage therapy is highly dependent on the efficiency and safety of phage preparations, which raises manufacturing and formulation challenges. The production of phages must comply with the strict regulations that are usually applied for pharmaceutical products to ensure the high-quality standards appropriate for their intended. This needs a production with a controlled and reproducible process. One of the requirements is to avoid phages encoding for lysogeny, virulence factors or antibiotic resistance. The presence of impurities such as endotoxins in phage preparations should also be avoided or be below a threshold. The presence/absence of neutralizing antibodies binding against phages must be known. The development of “phagogram” (in parallel to antibiogram) could be also an important way for the routine use of phage therapy. However, this could represent another approach for the treatment of infected wounds with minimal effects on the host microbiome (Hill et al., 2018; Morozova et al., 2018). Mendes et al. (2014) tested an *in vitro* cocktail of bacteriophages targeting *S. aureus, P. aeruginosa*, and *Acinetobacter baumannii* on both planktonic cells and biofilm-associated cells, and found that it reduced biofilm formation and infection. Other case reports have described encouraging results in patients with diabetic foot and chronic wounds (Fish et al., 2018; Taha et al., 2018).

To date, one of the main limitations is that the evaluation of phage efficiency has been performed using mainly *in vitro* studies and a single species in a biofilm. However, biofilms in DFU are multi-species, impacting the spatial organization and the interaction with phages. The specific outcome of phage infection in a multi-species biofilm seems to strongly depend on the bacterial species composing the biofilm (e.g., whether they establish synergist or antagonist interactions). The complexity of phage-biofilm interactions is increased by evidence of biofilm formation induced by exposure to certain phages (Lacqua et al., 2006; Tan et al., 2015; Henriksen et al., 2019). Overall, even if the potential of phages to control the complex biofilm observed in DFU is proved, the complexity and diversity of phage-biofilm interactions could limit broad conclusions and need more research to claim that phage therapy becomes a real solution in the DFU situation.

### Therapeutic Solutions on Wound Healing

Photodynamic therapy could be an interesting approach to aid wound healing. In this therapeutic procedure, pathogen cell death is induced upon exposure to light to generate oxygen species by activation of a photosensitizing agent. This agent is non-toxic in the dark, but after illumination, it becomes a very efficient antimicrobial agent. This method is used mainly in oncology, but it could also be employed to manage chronic wounds notably by its ability to prevent amputation in diabetic patients with DFU. Indeed, a clinical study showed that all non-treated patients (*n* = 16) underwent amputation, compared to only one patient in the group that received photodynamic therapy (*n* = 18) (Tardivo et al., 2014).

Another technology uses non-thermal plasma. Here, plasma is a partially ionized medium composed of many elements, such as charged particles (electrons and ions), neutral and excited atoms, UV photons and radicals. A recent study in rats showed that this technology could be used on pressure ulcers to accelerate wound healing (Chatraie et al., 2018). Additional investigations are needed to determine its value in humans, but recent results using an *in vivo* mouse model of type 2 diabetes showed promotion of bacterial killing of *P. aeruginosa* and wound disinfection without metabolic complication (Cooley et al., 2020).

Application of electrical stimulation has also been investigated in wound repair and regeneration. Wireless electroceutical dressings were recently tested in a porcine chronic wound polymicrobial biofilm infection model with *P. aeruginosa* (PAO1) and *A. baumannii* (19606) (Barki et al., 2019). The data suggested that the dressing disrupted wound biofilm aggregates and accelerated wound closure by restoring skin barrier function. The dressing changed expression of *P. aeruginosa* quorum sensing *mvfR* (*pqsR*), *rhlR*, and *lasR* genes and silencing of E-cadherin (a protein required for skin barrier function). Finally, this study highlighted the rescue effect against biofilm-induced persistent inflammation by decreased cytokines production.

Finally, hyperbaric oxygen therapy had been used for several years in the management of DFU. It consists in inhalation of pure oxygen after entering a special compression chamber. The treatment aims to increase the oxygen supply to the wound. However, the value of this therapy is controversial and its effect seems more due to the foot discharge than the oxygen itself. In a Cochrane review, the authors concluded that the therapy improved short-term but not long-term healing in patients with DFUs (Kranke et al., 2015).

### Alternatives in the Inhibition of Bacterial Adhesion and Biofilm

#### Inhibition of Initial Bacterial Adhesion

Bacterial growth requires the presence of metals (particularly, calcium, iron, and magnesium). Ionic chelators could be used to limit bacterial growth and initial adhesion. Ethylene diamine tetra-acetic (EDTA) and citrate are the most promising compounds of this class (Raad et al., 2008). However, the efficiency of these chelators is dependent on the bacterial strains. For instance, Abraham et al. (2012) observed that the anti-biofilm effect varied among *S. aureus* isolates. Aryl rhodanines also can inhibit the early stages of biofilm development by preventing the attachment on the surface of *S. aureus* and other Gram-positive bacteria, but not of Gram-negative bacteria (Opperman et al., 2009).

#### Inhibiting Biofilm Metabolism

Quorum Sensing (QS) is important for the transition from antimicrobial-sensitive planktonic cells to antimicrobial-resistant cell aggregates in a biofilm. In the absence of QS signal, biofilm formation is inhibited. Many researchers have evaluated compounds to modulate QS, such as furanone that inhibited, among others, *P. aeruginosa* biofilms (Kim et al., 2012), sodium ascorbate that modulated the QS signal in *P. aeruginosa* (El-Mowafy et al., 2014), savarin (a *S. aureus* virulence inhibitor) (Sully et al., 2014), and azithromycin in *P. aeruginosa* (Bala et al., 2011). Moreover, RNA III inhibiting peptide reduced *S. aureus* and *Staphylococcus epidermidis* virulence and improved healing in rats (Giacometti et al., 2003). These approaches are efficient only on a restricted number of bacterial species, and due to their potential toxicity, they have a limited use. Moreover, it has been reported that some bacteria isolated from clinical samples have become resistant to some QS modulators (García-Contreras et al., 2013), suggesting the emergence of multi-QS inhibitor resistant bacteria (Koul et al., 2016).

Another approach uses the cyclic diguanylate inhibition. Cyclic diguanylate (c-di-GMP) is a second messenger that controls many cellular functions, including biofilm formation. Various stress factors, such as starvation, reduced c-di-GMP level, leading to biofilm dispersal (Romling et al., 2013). This study also found that dispersed cells were more virulent compared with the first planktonic cells that induced the biofilm and with biofilm sessile cells. Moreover, small molecules, such as LP 3134, LP 3145, LP 4010, and LP 1062, inhibited a key enzyme that mediated c-di-GMP synthesis and consequently also biofilm formation in *P. aeruginosa* and *A. baumannii* (Wu et al., 2015). Unfortunately, these molecules seem to be toxic to eukaryotic cells. Finally, ebselen inhibited c-di-GMP and displayed good results on *P. aeruginosa* biofilms (Lieberman et al., 2014).

The biofilm matrix mainly contains proteins, extracellular DNA and polysaccharides. Polysaccharides are important for the early stage of biofilm formation and can protect cells during biofilm maturation. They also provide the basal biofilm structure that allows the bacterial community stratification. A recent study demonstrated that the exo-polysaccharide EPS273, obtained from a marine bacterium, reduced biofilm formation in *P. aeruginosa* by targeting virulence factors (Jiang et al., 2011). Other antibiofilm polysaccharides have been discovered, for instance Psl and Pel from *P. aeruginosa* PAO1 that decreased *S. epidermidis* biofilm formation in a co-culture biofilm *in vivo* model (Pihl et al., 2010). Other non-bacterial polysaccharides from animals, plants and algae have also shown antibiofilm activity (Rendueles et al., 2013).

In stress conditions, bacteria synthesize alarmones (guanosine tetraphosphate and guanosine pentaphosphate) called (p)ppGpp (Willcox et al., 2008). The antibiofilm peptide 1,018 inhibited their accumulation upon nutritional stress and prevented biofilm formation. Moreover, at low concentration, it eradicated biofilm-associated bacteria and disrupted mature biofilms. This peptide and its derivatives HE4 and HE10 were similarly effective against *P. aeruginosa* and *Burkholderia cenocepacia* biofilms (de la Fuente-Núñez et al., 2014). In addition, peptide 1,037 reduced biofilms formed by other bacteria (the Gram-negative pathogens *P. aeruginosa* and *B. cenocepacia*, and the Gram-positive *Listeria monocytogenes*) (de la Fuente-Núñez et al., 2012).

Finally, a recent study showed that the iron chelator deferiprone (DFP) affected bacterial biofilm formation and had synergistic effects (antibacterial activity) with some antibiotic compounds against coagulase-negative staphylococci. The potential of DFP is clearly based on its potentiation of the antibiotics action, leading to a significant biofilm reduction (Coraça-Huber et al., 2018).

#### Promoting Bacterial Dispersion

One promising therapeutic approach consists in targeting the EPS matrix with dispersing agents in combination with antibiotics. For instance, the α-amylase (Kalpana et al., 2012; Fleming et al., 2017) enzyme, which is produced by marine bacteria, can disrupt polysaccharide bonds. It is now used as a dispersing agent to target the polysaccharide bonds of the EPS matrix, leading to biofilm degradation *in vitro*. Other enzymes (deoxyribonuclease I, the hydrolases dispersin B and DNase) also showed EPS matrix-degrading properties (Fleming et al., 2017; Chen and Lee, 2018; Sharma and Pagedar Singh, 2018).

Some synthetic agents have been developed, such as 2-aminoimidazole for *S. aureus* biofilms (Rogers et al., 2010) and synthetic lysostaphin, an effective treatment for established biofilm infections on implanted jugular vein catheters in mice (Kokai-Kun et al., 2009).

In addition, the enzymes proteinase K and trypsin can eradicate biofilms from a variety of staphylococcal strains on inert surfaces. However, their efficacy for the elimination of established biofilms is not well known *in vivo*, thus limiting their therapeutic potential. Moreover, this strategy might lead to the release of bacteria from the biofilm into the blood circulation that could induce a strong inflammatory response or a systemic acute infection.

*Cis*-2-Decenoic acid (C2DA) is a fatty acid chemical messenger produced by *P. aeruginos*a that induces the dispersion of biofilms with *S. aureus* and other Gram-positive and Gram-negative bacteria (Davies and Marques, 2009). C2DA controls the initiation of biofilm formation and the dispersion of mature biofilms. C2DA can inhibit MRSA biofilm formation/growth, but cannot eradicate them (Jennings et al., 2012).

### New Generation of Dressing and Grafts

Parsons et al. (2016) developed a next-generation antibiofilm carboxymethylcellulose silver-containing wound dressing (NGAD). This hydrofiber dressing was designed to disperse the wound biofilm and to enhance ionic silver antimicrobial action. The authors showed that NGAD was more efficient (biofilm disruption and removal) than other commercial dressings in a large panel of clinical isolates, including *S. aureus* and antibiotic-resistant *P. aeruginosa*. Another *in vitro* study conducted on a novel wound-dressing material based on a matrix of the polysaccharides alginate, hyaluronic acid and Chitlac-silver nanoparticles concluded that hyaluronic acid was able to stimulate the wound healing simultaneously to the silver particles allowing efficient antibacterial activity against biofilms (Tarusha et al., 2018). Pérez-Díaz et al. (2018) combined these nanoparticles with mesenchymal stem cells (MSC) that can improve wound healing due to their ability to differentiate and release growth factors. In addition to the MSC and nanoparticles, they used radiosterilized pig skin as a matrix to deliver MSC into wound beds. *In vitro* data suggested a decrease of bacterial growth and biofilm formation. Finally, Ramalingam et al. (2019) conducted a study presenting the utility of electrospun nanofiber containing a natural extract (Gymnema sylvestre) that prevented biofilm formation, inhibited both Gram-positive and Gram-negative bacteria, and enhanced human dermal fibroblasts development in an *in vitro* model.

A surfactant-based wound gel dressing described in a porcine skin explant infected with *P. aeruginosa* (PAO1) biofilm showed encouraging results. Dressing the wound with this gel reduced bacteria development and biofilm infection (Yang et al., 2017). More recently, Percival et al. (2018) highlighted the effects of a non–ionic surfactant, the Pluronic F127 used in combination with melatonin and chitosan in a wound dressing. The microspheres of Pluronic F127 enhanced chitosan properties allowing antimicrobial and antibiofilm activity against *S. aureus* (Percival et al., 2018).

A dehydrated amniotic membrane allograft was used in 22 patients with lower extremity wounds (Lullove, 2017). At week 12 after application of the human amniotic membrane, DFU were completely healed.

A randomized double-blind clinical trial showed that sucrose octasulfate significantly improves wound closure in neuroischemic DFU after 20 weeks of treatment (Edmonds et al., 2018).

Skin substitutes could be another therapeutic solution in wound healing. The Food and Drug Administration recently approved a treatment for wound care involving fish skin after a clinical trial to determine its effectiveness on burns and different wound types. Fish skin contains omega-3 fatty acids that have natural anti-inflammatory properties and can accelerate healing. An ongoing clinical trial is evaluating an extracellular matrix that binds to the cells around the wound and promotes the growth of new skin (see clinicaltrials.gov/NCT01348581).

*Arenicola marina* is a technology based on the finding that lack of oxygen in chronic wounds hampers healing and cell proliferation (Le Pape et al., 2018). HEMHealing^®^ (Hemarine) provides oxygenation to the wound by including M101 hemoglobin in the dressing matrix. M101 hemoglobin is an oxygen carrier that belongs to the extra-cellular hemoglobin family and is found in the *Arenicola marina* marine worm. This hemoglobin can naturally fix oxygen from the external environment and then gradually release it in the hypoxic medium to restart the healing process. In a wound context, it could restart cell proliferation and decrease wound budding.

Skin works as an extracellular matrix that binds to the cells around the wound and promotes the growth of new skin. Epigel^®^ (Epinova Biotech) is an innovative patch based on a highly hydrophilic, biocompatible and bioactive hydrogel scaffold that supports wound bed hydration, thus reducing healing time. Clinical trials must be done to evaluate the value of Epigel^®^ as wound dressing.

Hwang et al. (2019) evaluated allogeneic keratinocyte grafts (weekly grafts for up to 12 weeks) in 71 patients with intractable DFUs. They reported wound healing in 78.8% of patients: 64.7% with complete healing within an average of 6.1 weeks, and 14.1% with partial healing and an average 35.5% reduction of the initial size at the end of the follow up. This treatment seems effective for chronic and difficult-to-treat DFUs. In line with other studies [in fetal cells (Momeni et al., 2019) or epithelial cells (Lo et al., 2019)] in other wound types, this study showed the benefit of skin grafts that could represent the future management of chronic wounds. Indeed, progenitor stem cells present in these grafts can accelerate wound repair and tissue regeneration, and consequently decrease the risk of wound infection. A significant number of stem cell therapies for cutaneous wounds are currently under development (Kanji and Das, 2017).

Chronic wounds are inflammatory processes that result in the increase of proteolytic enzymes and degradation of the extracellular matrix. Two studies investigated the impact of providing a biocompatible scaffold to support the healing. They used a purified Type I collagen matrix containing polyhexamethylene biguanide on patients (*n* = 8 and *n* = 41) suffering from DFU. Their results suggested that the collagen matrix improved both wound closure and the wound bed condition (Lintzeris et al., 2018; Oropallo, 2019). Finally, a study investigated the effect of human placental tissues against *P. aeruginosa* and *S. aureus* biofilm (Mao et al., 2018). It highlighted the fact that both human cryopreserved viable amniotic membrane and cryopreserved viable umbilical tissue had antibacterial activity against multiple bacterial pathogens and demonstrated that these tissues released factors that inhibited biofilm formation of *P. aeruginosa* and *S. aureus* in an *ex vivo* porcine model. Recently, use of adipose-derived stem cell improved wound healing by promoting angiogenesis and/or vascularization, modulating immune response, and inducing epithelialization in the wound (Hassanshahi et al., 2019).

The effectiveness of 3D-printed scaffolds in chronic wounds has not yet been proven, but this seems to be a promising strategy. Sun et al. (2018) reported that 3D-printed scaffold membrane alone (*n* = 1 patient), and 3D-printed scaffold powder mixed with platelet-rich fibrinogen (*n* = 2) reduced healing time in patients with pressure ulcer and/or DFU (Sun et al., 2018). Another group developed a 3D-printed scaffold that included a drug delivery system based on the body temperature (Pushparaj and Ranganathan, 2017). Although this device has not been tested *in vivo* yet, it is the first step toward the use of 3D-printed scaffolds that incorporate the delivery of drugs (antibiotics or antibiofilm molecules) to shorten healing time and decrease the risks of infection and complication.

## Conclusion

The severity of DFU and the difficulty in treating it has prompted researchers to take a closer look at these infections and the associated issues. Biofilms play a crucial role in DFUs and contribute to delay healing. Research now must take into account the biofilm bacterial organization in these chronic wounds in order to identify novel alternative therapeutic candidates to improve DFU management. As we described above, alternative strategies such as bacteriophages, probiotics, AMPs or antibiofilms are exciting strategies and show promising results. All these compounds could provide solutions against MDR bacteria. However, additional studies are required to understand the biofilm bacterial organization in DFU, and also the mechanisms behind each of the candidates to improve the wound healing management and thus offer new therapeutic solution for the management of DFU.

## Author Contributions

CP, PL, J-PL, and AS wrote the manuscript. CD-R, AP, AB-D, and SS critically reviewed the manuscript. All authors contributed to the article and approved the submitted version.

## Conflict of Interest

CP is the recipient of a grant from Biofilm Control (Bourse CIFRE). The remaining authors declare that the research was conducted in the absence of any commercial or financial relationships that could be construed as a potential conflict of interest.

## Publisher’s Note

All claims expressed in this article are solely those of the authors and do not necessarily represent those of their affiliated organizations, or those of the publisher, the editors and the reviewers. Any product that may be evaluated in this article, or claim that may be made by its manufacturer, is not guaranteed or endorsed by the publisher.

## Abbreviations

AMP: antimicrobial peptide
c-di-GMP: cyclic diguanylate
C2DA: *cis*-2-decenoic acid
DFI: diabetic foot infection
DFP: deferiprone
DFU: diabetic foot ulcer
DNA: deoxyribonucleic acid
DMSO: dimethyl sulfoxide
EDTA: ethylene diamine tetra-acetic
EGTA: egtazic acid
EPS: extracellular polymeric substance
FEP: functionally equivalent pathogroups
MDR: multidrug resistance
MRSA: methicillin-resistant *Staphylococcus aureus*
MSC: mesenchymal stem cells
NGAD: next generation antibiofilm carboxymethyl cellulose silver containing wound dressing
QS: quorum sensing.
